# Tracking the changes in longitudinal MRI-detected perivascular spaces following ischaemic stroke

**DOI:** 10.1093/esj/aakag108

**Published:** 2026-09-07

**Authors:** William Pham, Mohamed S Khlif, Zhibin Chen, Alexander Jarema, Luke A Henderson, Vaughan G Macefield, Amy Brodtmann

**Affiliations:** Department of Neuroscience, School of Translational Medicine, Faculty of Medicine, Nursing and Health Sciences, Monash University, Melbourne, Victoria, Australia; Department of Neuroscience, School of Translational Medicine, Faculty of Medicine, Nursing and Health Sciences, Monash University, Melbourne, Victoria, Australia; Department of Neuroscience, School of Translational Medicine, Faculty of Medicine, Nursing and Health Sciences, Monash University, Melbourne, Victoria, Australia; Department of Radiology, Alfred Health, Melbourne, Victoria, Australia; School of Medical Sciences (Neuroscience), Brain and Mind Centre, The University of Sydney, Sydney, New South Wales, Australia; Department of Neuroscience, School of Translational Medicine, Faculty of Medicine, Nursing and Health Sciences, Monash University, Melbourne, Victoria, Australia; School of Medicine, Western Sydney University, Sydney, New South Wales, Australia; Department of Neuroscience, School of Translational Medicine, Faculty of Medicine, Nursing and Health Sciences, Monash University, Melbourne, Victoria, Australia

**Keywords:** biomarkers, cerebral small vessel disease, ischaemic stroke, MRI, perivascular spaces, Virchow–Robin spaces

## Abstract

**Introduction:**

Stroke is a leading cause of mortality and morbidity worldwide. Magnetic resonance imaging-visible perivascular spaces (PVS) are an emerging marker of cerebral small vessel disease and may have prognostic value in stroke. We aimed to characterise longitudinal changes in PVS volume and cluster count following ischaemic stroke.

**Patients and methods:**

Perivascular space volumes and cluster counts were compared between stroke survivors (*n* = 124; 39 women; median [Q1, Q3] age = 70 [62, 76] years) and healthy controls (*n* = 39; 15 women; median age = 69 [66, 72.5] years). Magnetic resonance imaging scans were acquired at 3 months, 12 months and 36 months post-stroke. Perivascular spaces were automatically segmented from T1-weighted MRI using a validated deep learning algorithm (nnU-Net). Generalised linear mixed-effects models were used to assess group differences and longitudinal changes in PVS, adjusting for baseline age, sex, total intracranial volume and body mass index.

**Results:**

At the 3-month timepoint, whole-brain PVS metrics were significantly greater in the stroke group than in controls. By 36 months, stroke patients demonstrated significant PVS reductions relative to controls in the frontal lobe (PVS volume: exp(β) = 0.93; 95% CI, 0.82, 0.99, *P* = .032; PVS cluster count: exp(β) = 0.91; 95% CI, 0.83, 1; *P* = .037).

**Discussion:**

Contrary to age-related PVS enlargement observed in controls, stroke survivors showed significant longitudinal reductions in PVS volume and cluster counts over 36 months. These findings suggest that MRI-visible PVS trajectories after stroke are dynamic and region-specific, challenging the interpretation of PVS enlargement as a simple marker of cerebrovascular disease burden.

**Conclusion:**

Ischaemic stroke is associated with dynamic and regional changes in PVS volume and count.

## Graphical Abstract

**Graphical Abstract:**
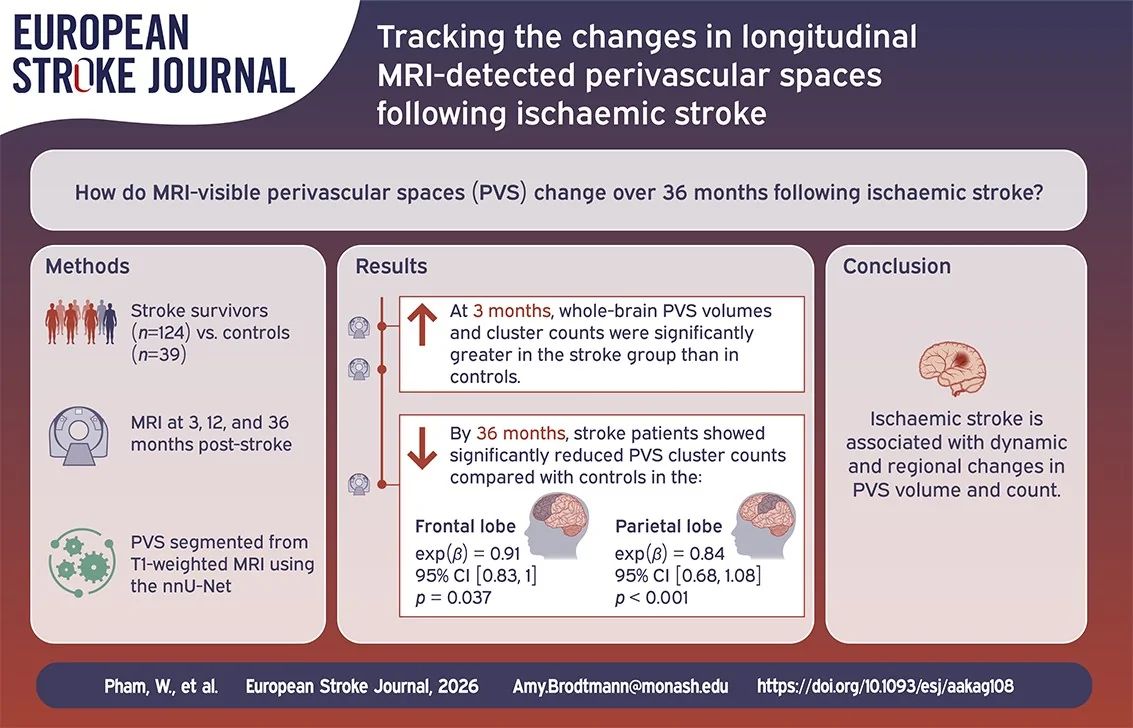

## Introduction

Ischaemic stroke accounts for 71% of all stroke cases and is a leading cause of worldwide mortality and morbidity.^1^^,^^2^ Ischaemic stroke is associated with accelerated brain-wide atrophy, cortical thinning and white matter neurodegeneration.^3–6^ These structural changes evolve over months to years following stroke.

Perivascular spaces (PVS) are fluid-filled spaces surrounding cerebral blood vessels. According to the Standards for Neuroimaging Research in Small Vessel Disease (STRIVE) criteria, enlarged PVS are identified as key MRI markers of cerebral small vessel disease (CSVD) along with lacunar infarctions, white matter hyperintensities (WMHs), cerebral microbleeds and cortical superficial siderosis.^7^ Individuals with CSVD are at elevated risk of both ischaemic stroke and subsequent cognitive decline.^8^ Magnetic resonance imaging-visible PVS are increasingly regarded as non-invasive biomarkers of CSVD, with enlargement linked to ageing and cerebrovascular pathology.^9^^,^^10^

Perivascular spaces also function as critical pathways for cerebrospinal fluid movement and are postulated to have a role in waste clearance within the putative human glymphatic system.^11^ Animal studies have shown that the perivascular fluid flow along the glymphatic system is a major driver of cerebral oedema post-ischaemia.^12^ Moreover, impaired glymphatic function after ischaemic stroke has been linked to the deposition of amyloid-β oligomers and secondary neuronal injury in the ipsilateral thalamus.^13^

Traditionally, investigations of perivascular space enlargement in MRI relied on visual rating, where enlargement scores are assigned based on the number of visible PVS clusters in selected axial slices and on predefined thresholds.^14^ Accordingly, PVS enlargement in the centrum semiovale (CS) has been identified as a predictor of early mortality in haemorrhagic stroke.^15^ Whereas, basal ganglia (BG) PVS counts are associated with an increased risk of haemorrhagic rebleeding.^16^ No significant associations between PVS burden in the BG or CS and the risk of ischaemic stroke recurrence were found in one meta-analysis.^17^ This is possibly due to visual rating of PVS being suboptimal, reliant on predefined slices and subjective assessments, and therefore fails to capture the full extent of PVS enlargement.

Recent advances in MRI segmentation algorithms have enabled voxel-wise quantification of PVS throughout the entire brain.^18^^,^^19^ In this study, we utilised our own deep learning models to automatically quantify PVS (volume and cluster count) across several brain regions based on T1-weighted (T1w) MRI scans.^20^ We aimed to track the longitudinal PVS changes in ischaemic stroke survivors scanned 3, 12 and 36 months post-stroke. We hypothesised that stroke patients would exhibit greater PVS burden than stroke-free controls, with greater PVS enlargement (ie, increased counts and volume) over time.

## Patients and methods

The *Cognition and Neocortical Volume After Stroke* (CANVAS) study is a prospective observational cohort study comprising individuals with ischaemic stroke in any site or territory (*n* = 124) and age- and sex-matched controls without a history of stroke (*n =* 39).^21^ The sample sizes for the control and stroke cohorts were determined a priori and have been reported previously.^21^^,^^22^

Participants were recruited from 3 hospitals in Victoria, Australia: Austin Health, Box Hill Hospital and the Royal Melbourne Hospital. Stroke patients were enrolled from the Acute Stroke Units at each site, with diagnosis confirmed through clinical neuroimaging (CT or MRI). Exclusion criteria for both groups included a history of cognitive impairment, inability to undergo MRI scanning or a diagnosis of a severe medical illness. Assessments were conducted at 5 timepoints: baseline < 6 weeks of stroke, 3-, 12-, 36- and 60-month post-stroke.

The study was approved by respective Human Research Ethics Committees in participating institutions. Informed written consent was obtained from all participants in accordance with the Declaration of Helsinki.

### Participants

We included participants with available data at 3-, 12- and 36-month timepoints for this analysis. Demographic and clinical information were collected including age, sex, body mass index (BMI), vascular risk factors and comorbidities.

### Imaging protocol

Whole-brain imaging was performed using a 3T Siemens Tim Trio scanner equipped with a 12-channel head coil (Siemens, Erlangen, Germany). T1-weighted images were acquired using a magnetisation-prepared rapid acquisition gradient echo (MPRAGE) sequence with the following parameters: TR = 1900 ms; TE = 2.55 ms; TI = 900 ms; flip angle = 9°; FOV = 256 × 256; and voxel size = 1.0 mm isotropic. All scans were manually reviewed and images affected by motion artefacts or other sources of degradation were excluded. Stroke lesion locations were determined per study protocol.^21^ However, these data were not incorporated into the present analyses, and stroke lesion characteristics were not accounted for in this study.

### Perivascular space quantification

Perivascular spaces were automatically segmented in T1w MRI scans using a previously validated convolutional neural network (nnU-Net).^20^^,^^23^ As the nnU-Net models were trained on T1w MRI, which was more consistently available than T2-weighted (T2w) MRI, analyses were restricted to T1w images. The model was developed and validated using manual PVS segmentations generated by an experienced rater and reviewed by a radiologist, with full methodological details reported in our previous study.^20^

The model produced segmentation maps with distinct voxel-wise labels for PVS in the white matter and BG. Midbrain and hippocampal PVS were automatically quantified by dedicated nnU-Net models.^20^ The 2 PVS metrics considered in this study were volumes and cluster counts. Perivascular space enlargement was defined as an increase in either PVS volume or count, and PVS shrinkage as a decrease in either metric. Perivascular space cluster counts were determined using the “*measure.label*” function from the “scikit-image” Python package,^24^ which identifies spatially distinct PVS clusters within the segmentation mask. All PVS segmentation, processing and quantification were conducted using Python (v3.9.13).

#### Quantification via region and arterial territory

Perivascular space metrics were further quantified within specific regions of interest (ROIs). A total of seven ROIs were defined based on 2 brain atlases. Lobar regions, including the frontal, parietal, temporal and occipital lobes, were defined according to the ICBM2009 asymmetrical atlas.^25^ Additionally, PVS metrics were quantified within supratentorial cerebral arterial territories, including the anterior cerebral artery (ACA), MCA, and posterior cerebral artery (PCA) regions, as defined by a vascular territory atlas.^26^ Parcellating PVS metrics across brain regions enables the assessment of spatial heterogeneity, allowing us to determine whether ischaemic stroke is associated with region-specific alterations in PVS.

**Figure 1:**
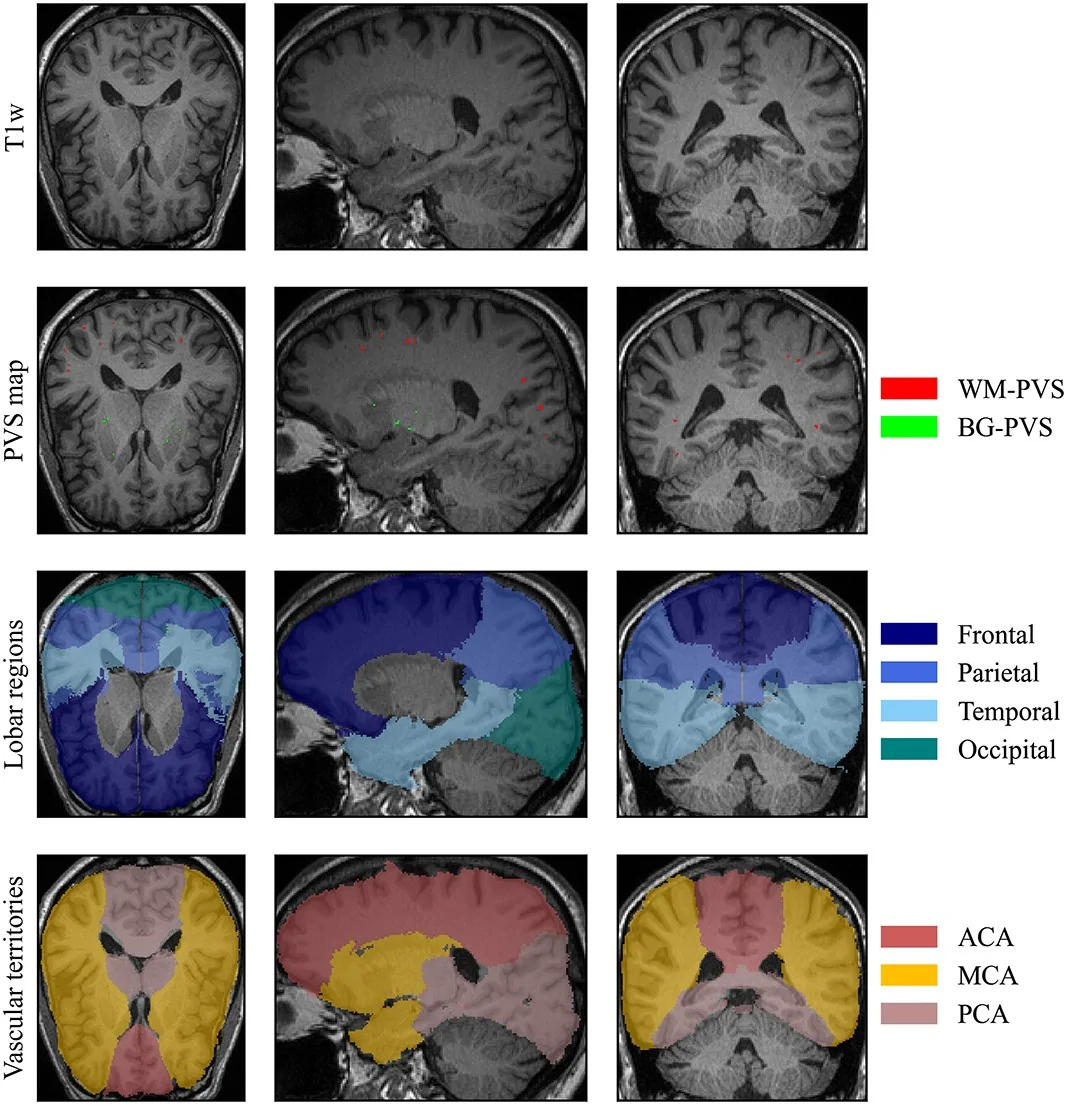
Examples of T1w MRI with segmented PVS maps and region atlas parcellations according to lobar regions and arterial vascular territories. Top row: Raw T1w MRI scan from a stroke-free participant with images from the axial, sagittal, and coronal planes. Second row: PVS map overlaid on T1w MRI, with WM-PVS and BG-PVS. Third row: Lobar regions including the frontal, parietal, temporal and occipital lobes. Bottom row: Arterial vascular territories including the regions supplied by the ACA, MCA and PCA. Abbreviations: ACA = anterior cerebral artery; BG = basal ganglia; PCA = posterior cerebral artery; PVS = perivascular spaces; T1w = T1-weighted; WM = white matter.

While the lobar regions reflect the functional organisation at a whole-brain level, vascular territories delineate areas with distinct blood perfusion supplied by major arterial branches. Substantial overlap exists between these 2 parcellations (Figure 1). The ACA supplies the medial portions of the frontal and parietal lobes, the MCA perfuses the lateral aspects of all 4 lobes and the PCA primarily supplies the occipital lobe and posterior temporal lobe.

T1w MRI scans were further processed using FastSurfer (v2) to extract baseline total intracranial volume (TIV) and generate brain parcellations for each subject.^27^^,^^28^ Extracted brain volumes were spatially registered to the MNI152 template using Advanced Normalization Tools (ANTs, v2.4.3).^29^ Registration was performed with antsRegistrationSyn.sh (rigid, affine, symmetric diffeomorphic normalisation), producing forward and inverse transforms. Subject-specific atlases were generated by applying the inverse warp fields and affine transformations to the ICBM2009c atlas and arterial territories atlas.

Perivascular space volumes and cluster counts were computed for each ROI by overlaying the subject-specific atlases with corresponding PVS segmentation maps. A PVS cluster was considered to belong to a given ROI if its centroid was situated within that ROI. The centroid was determined with the scikit image “*measure.centroid*” function. The nnU-Net model was trained to specifically segment PVS and not stroke lesions.

All image processing was performed on the MASSIVE High Performance Computing cluster.^30^ Perivascular space segmentation using nnU-Net was conducted on an NVIDIA A40 GPU equipped with 256 GB RAM.

### Statistical analysis

To assess the reliability of the automated PVS measurements, a validation procedure was conducted using manual PVS counts as the reference standard. In the baseline T1w MRI of all subjects, PVS were manually counted in 2 representative axial slices in the CS and BG. In the BG, one axial slice was positioned at the level of the anterior commissure and the second axial slice superior to the anterior commissure. Perivascular spaces were also manually counted throughout the entire midbrain and hippocampal regions. For the CS and BG, automated PVS counts were extracted from the same axial slices used for manual rating, while full-region comparisons were performed for the midbrain and hippocampi.

We computed Lin’s concordance correlation coefficient (CCC) to evaluate the agreement between manual and automated PVS counts, and Spearman’s rank correlation coefficient to assess the monotonic association between the 2 methods. Perivascular space validation was conducted on all MRI scans (Table SS1). Manual annotations were performed by an experienced rater (W.P.) and independently reviewed by a board-certified radiologist (A.J.).

We assessed group differences in PVS metrics and their longitudinal changes using generalised linear mixed-effects models (GLMMs). Since most PVS metrics were not normally distributed, we modelled them using a Tweedie distribution with a log link function.

Generalised linear mixed-effects models with the Tweedie distribution demonstrated good model fit for all PVS metrics, except for those in the midbrain and hippocampus. Consequently, PVS metrics in the midbrain and hippocampus were binarised into “high” and “low” categories based on a median cut-off. Binary outcomes were modelled using GLMMs with binomial family and log-link functions to evaluate the effects of stroke diagnosis and the group × timepoint interaction term.

All models included PVS metrics as the dependent variable, with timepoint × group interaction as a fixed effect, and a random intercept for each subject to account for intra-individual differences. Baseline age, sex, TIV and BMI were included as covariates in all models as these factors have previously been associated with PVS measurements.^31^^,^^32^ Since previous studies have shown age-related changes in PVS to be non-linear, baseline age was modelled using a second-order spline term.^10^^,^^33^

For all GLMMs, the normality of residuals was assessed by visually inspecting Q-Q plots, and homoscedasticity was assessed by inspecting the residual plots. Model diagnostics included visual inspection of residuals, assessment of overdispersion and zero-inflation using the “*simulateResiduals*” function from the *DHARMa* package, and comparison of Akaike information criterion and Bayesian information criterion (BIC) for model selection. Covariates were removed if inclusion compromised residual normality or homoscedasticity.

To derive robust estimates, 95% CIs for fixed effects were calculated using parametric bootstrapping with 1,000 simulations. Statistical significance was assessed based on the adjusted *P*-values (*P_adj_*) with an α level of 0.05. All GLMMs were implemented using the *glmmTMB* package in R (v4.2.1). To account for multiple comparisons, *P*-values from all models were adjusted using the Benjamini–Hochberg false discovery rate correction.

## Results

One hundred and sixty-three participants (54 females, median [Q1, Q3] age = 69 [63, 75] years) had available data: 124 stroke survivors (stroke group) and 39 stroke-free controls (control group). Groups were balanced for age and sex, but the controls had significantly more years of education and, unsurprisingly, the stroke group had more vascular risk factors. Across the cohort, median [Q1, Q3] total PVS volume and cluster counts at baseline were 1,655 [1,094, 2,263] mm^3^ and 153 [108, 205.5], respectively. There was no significant difference in baseline PVS volume and cluster counts between groups. Subject demographics, MRI availability at each timepoint and clinical characteristics are summarised in Table 1. Clinical characteristics of the stroke cohort, including Oxfordshire stroke subtype, stroke laterality, NIHSS and mRS scores and stroke classification (recurrent vs first-ever), are summarised in Table 2.

**Table 1:** Baseline demographic and clinical characteristics of the study cohort. For normally distributed numerical variables, values are presented as mean ± SD. For non-normally distributed numerical variables, values are presented as median [Q1, Q3]. Categorical variables were compared using the chi-squared test, and continuous variables using Mann–Whitney *U* tests.

Characteristic	Control (*n* = 39)	Stroke (*n* = 124)	Total (*n* = 163)	*P*-value
**Age (years)**	69 [66, 72.5]	70 [62, 76]	69 [63, 75]	.8
**Sex, *n* female (%)**	15 (38.5%)	39 (31.5%)	54 (33.1%)	.42
**MRI availability, *n***				
** 12-month**	38	111	149	
** 36-month**	38	95	133	
**Education (years)**	17 [11, 18]	12 [10, 15]	13 [10, 16]	<.001
**Baseline TIV (cm** ^**3**^ **)**	1,471.8 ± 112.9	1,449.3 ± 155.6	1,454.7 ± 146.5	.41
**BMI**	26.6 ± 3.8	27.7 ± 4.4	27.4 ± 4.2	.06
**Composite VRFs, *n* (%)**	16 (41%)	87 (70.2%)	103 (63.2%)	<.001
**PVS volume (mm** ^**3**^ **)**	1,469 [989, 2,020]	1,727.5 [1,134.3, 2,493.3]	1,655 [1,094, 2,263]	.09
**PVS clusters**	144 [93.5, 178.5]	159.5 [109.5, 218.5]	153 [108, 205.5]	.1

Abbreviations: BMI = body mass index; PVS = perivascular space; TIV = total intracranial volume; VRFs = vascular risk factors.

**Table 2:** Clinical characteristics of the stroke cohort across baseline, 12-month and 36-month follow-up assessments. Data are presented as *n* (%). Oxfordshire stroke subtype, side of stroke, NIHSS, mRS and stroke classification (RS vs FE) are shown for participants with available data. Variables were compared using the chi-squared test or Fisher’s exact test, as appropriate.

	Baseline (*n* = 124)	12 M (*n* = 111)	36 M (*n* = 95)	*P*-value
**Oxfordshire type, *n* (%)**				1
**LACI**	17 (13.7%)	15 (13.5%)	12 (12.6%)	
**POCI**	42 (33.9%)	39 (35.1%)	31 (32.6%)	
**PACI**	64 (51.6%)	56 (50.5%)	51 (53.7%)	
**TACI**	1 (0.8%)	1 (0.9%)	1 (1.1%)	
**Side of stroke *n* (%)**				1
** Left**	48 (38.7%)	43 (38.7%)	38 (40.0%)	
** Right**	73 (58.9%)	65 (58.6%)	54 (56.8%)	
** Bilateral**	3 (2.4%)	3 (2.7%)	3 (3.2%)	
**NIHSS, *n* (%)**				.22
** NA**	3 (2.4%)	–	–	
** 0**	63 (50.8%)	78 (70.3%)	69 (72.6%)	
** 1**	27 (21.8%)	14 (12.6%)	13 (13.7%)	
** 2**	12 (9.7%)	9 (8.1%)	7 (7.4%)	
** 3**	8 (6.5%)	3 (2.7%)	2 (2.1%)	
** 4**	5 (4%)	2 (1.8%)	2 (2.1%)	
** 5**	2 (1.6%)	3 (2.7%)	1 (1.1%)	
** 6**	1 (0.8%)	1 (0.9%)	0 (0%)	
** 7**	3 (2.4%)	0 (0%)	1 (1.1%)	
** 8**	0 (0%)	1 (0.9%)	0 (0%)	
**mRS score, *n* (%)**				.18
** NA**	2 (1.6%)	–	–	
** 0**	24 (19.4%)	34 (30.6%)	34 (35.8%)	
** 1**	61 (49.2%)	56 (50.5%)	41 (43.2%)	
** 2**	25 (20.2%)	17 (15.3%)	16 (16.8%)	
** 3**	9 (7.3%)	3 (2.7%)	3 (3.2%)	
** 4**	3 (2.4%)	1 (0.9%)	1 (1.1%)	
**RS vs FE, *n* (%)**				.94
** RS**	19 (15.3%)	16 (14.4%)	13 (13.7%)	
** FE**	105 (84.7%)	95 (85.6%)	82 (86.3%)	

Abbreviations: FE, first-ever; LACI, lacunar infarct; PACI, partial anterior circulation infarct; POCI, posterior circulation infarct; RS, recurrent stroke; TACI, total anterior circulation infarct.

### Regional validation of automated perivascular space quantification

Agreement between manual and automated PVS counts was evaluated in selected axial slices for the CS and BG, as well as across the entire midbrain and hippocampal regions. Strong agreement was observed in the CS, with a high correlation and concordance between manual and predicted counts (Spearman’s ρ = 0.84; *P* < .001; CCC = 0.76; 95% CI, 0.73, 0.79). Moderate agreement was found for the BG-PVS counts (Spearman’s ρ = 0.67; *P* < .001; CCC = 0.57; 95% CI, 0.53, 0.61). The midbrain PVS counts showed weak correlation and low concordance between manual and predicted counts (Spearman’s ρ = 0.54; *P* < .001; CCC = 0.53; 95% CI, 0.41, 0.63), while the hippocampal PVS counts demonstrated moderate correlation and agreement (Spearman’s ρ = 0.72; *P* < .001; CCC = 0.68; 95% CI, 0.6, 0.74). Notably, these patterns of agreement were consistent across both stroke patients and controls.

In the CS, the model demonstrated consistently strong agreement across all time points and both diagnostic groups, with Spearman’s correlation coefficients exceeding 0.80 and Lin’s CCCs exceeding 0.70 (Table SS2). The finding indicates that the model maintains stable performance throughout the follow-up scans. Agreements in the BG and hippocampus were more modest, consistent with our previous validation study. Across the timepoints, midbrain PVS counts showed low to moderate agreement (Lin’s CCC: 0.45–0.71). Nevertheless, model performance remained comparable across all time points and diagnostic groups (Table S2).

### Global perivascular space comparison

**Figure 2:**
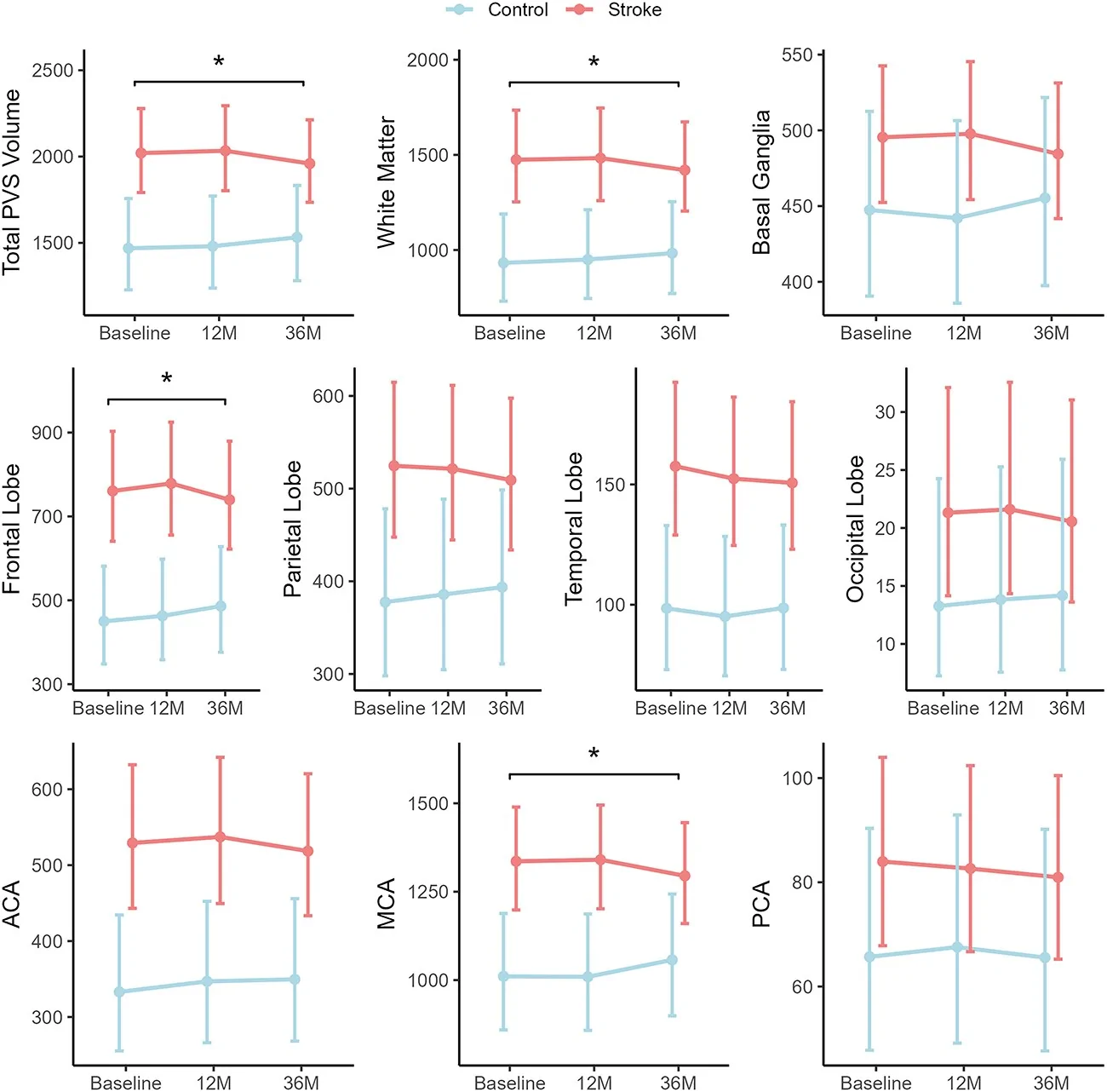
Longitudinal changes in predicted perivascular space volumes by brain region and arterial territory in stroke patients versus controls. Line plots display estimated marginal means (±95% CI) from generalised linear mixed effects models for total, regional and vascular territory PVS volumes across 3 timepoints: baseline, 12 months and 36 months. Significant group effects and Group × Time (36 M) interaction effects are annotated in each panel with corresponding exp(β) estimates and bootstrapped 95% CIs. Significance: ^*^P < .05, ^**^P < .01, ^***^P < .001. Significant PVS volume reductions were observed in stroke patients at the 36-month timepoint across the whole-brain and in several regions including the white matter, frontal lobe and middle cerebral artery territory. However, none of these associations remained statistically significant after correction for multiple comparisons (*P_adj_* > .05). Abbreviations: ACA = anterior cerebral artery; PCA = posterior cerebral artery; PVS = perivascular spaces.

**Figure 3:**
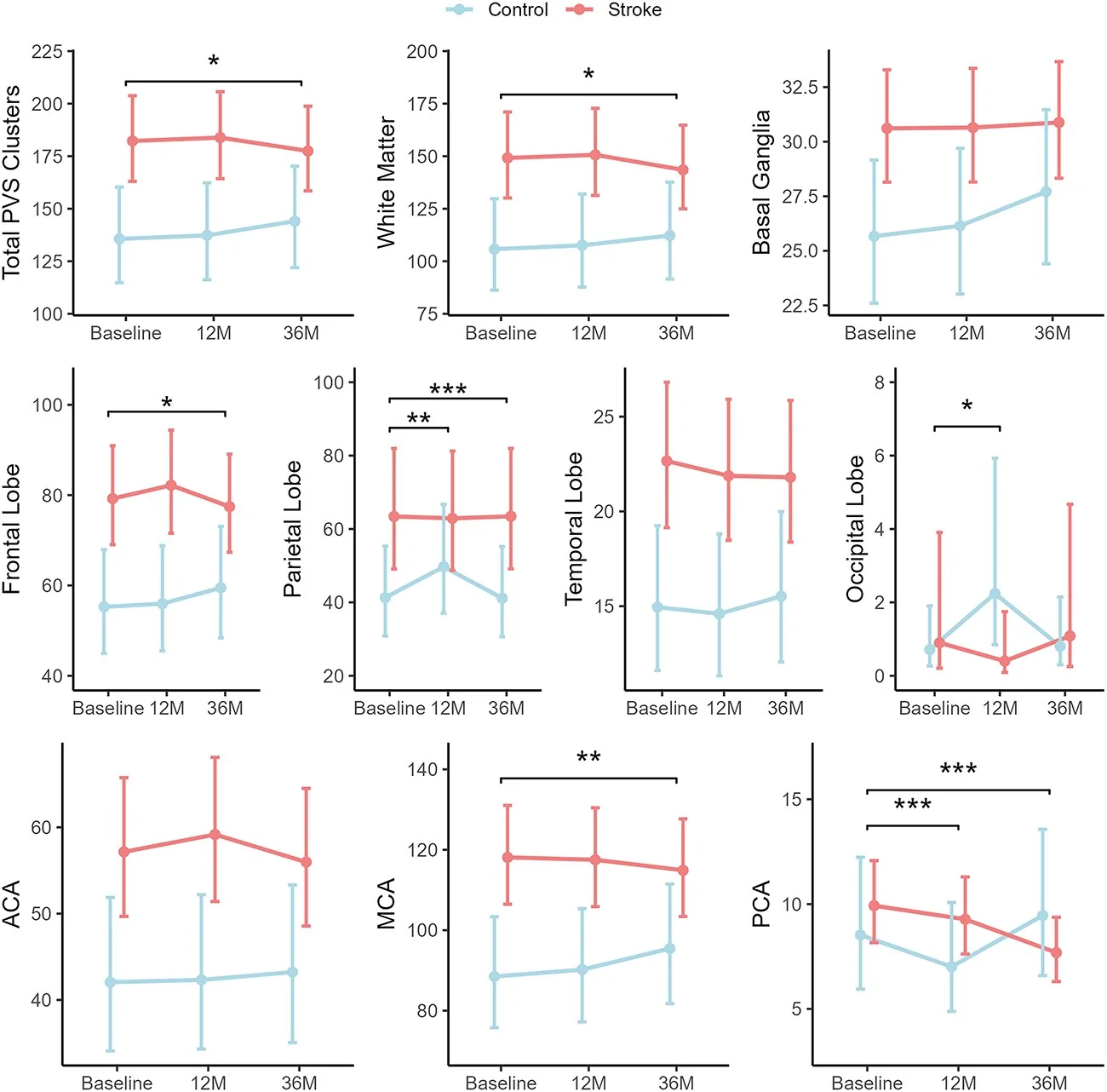
Longitudinal changes in predicted perivascular space cluster counts by brain region and arterial territory in stroke versus control groups. Line plots display estimated marginal means (±95% CI) from generalised linear mixed-effects models for total, regional and vascular territory PVS cluster counts across 3 timepoints: baseline, 12 months and 36 months. Significant group effects and Group × Time (36 M) interaction effects are annotated in each panel with corresponding exp(β) estimates and bootstrapped 95% CIs. Significance: ^*^P < .05, ^**^P < .01, ^***^P < .001. Significant reductions in PVS cluster counts were observed in stroke patients at the 36-month timepoint across the whole-brain and in several regions including the white matter, frontal and parietal lobes and middle and posterior cerebral artery territories. After correcting for multiple comparisons, associations in the parietal lobe and the middle and posterior cerebral artery territories remained statistically significant (*P_adj_* < .05). Abbreviations: ACA = anterior cerebral artery; PCA = posterior cerebral artery; PVS = perivascular spaces.

At baseline, the median total PVS volume was higher in stroke patients (1,723 [1,134.3, 2,493.3] mm^3^) than in controls (1,451 [989, 2,020] mm^3^). After adjusting for covariates, the stroke group exhibited greater total PVS volume (exp(β) = 1.38; 95% CI, 1.13, 1.69; *P* = .003; *P_adj_* = .015) and total PVS cluster counts (exp(β) = 1.34; 95% CI, 1.09, 1.63; *P* = .003; *P_adj_* = .015). While controls exhibited progressive enlargement of whole-brain PVS volume and cluster counts across the 3 years, stroke patients demonstrated a trend toward shrinkage over the same period. At 36 months, the stroke group showed nominally greater reductions in both PVS volume (exp(β) = 0.93; 95% CI, 0.87, 1; *P* = .035; *P_adj_* = .092) and cluster counts (exp(β) = 0.92; 95% CI, 0.85, 0.99; *P* = .02; *P_adj_* = .063). However, these associations were not statistically significant after correcting for multiple comparisons. No significant between-group effects were observed at the 12-month timepoint for any global or regional PVS metric (all *P* > .05). All model results are illustrated in Figures 2 and 3.

### Perivascular spaces in the white matter and basal ganglia

In the white matter, both PVS volume (exp(β) = 1.58; 95% CI, 1.19, 2.1; *P* = .001; *P_adj_* = .009) and cluster counts (exp(β) = 1.41; 95% CI, 1.1, 1.82; *P* = .004; *P_adj_* = .018) were significantly higher in the stroke group. Over 36 months, the stroke group exhibited nominally greater reductions in WM PVS volume (exp(β) = 0.91; 95% CI, 0.84, 0.99; *P* = .037; *P_adj_* = .092) and cluster counts (exp(β) = 0.91; 95% CI, 0.83, 0.98; *P* = .021; *P_adj_* = .063) across the 36-month period compared to controls. However, these associations did not meet the required significance threshold after accounting for multiple comparisons.

In contrast, there was no significant group effect for BG PVS volume (exp(β) = 1.11; 95% CI, 0.94, 1.29; *P* = .21), nor significant group-by-time interaction at the 36-month timepoint (exp(β) = 0.96; 95% CI, 0.9, 1.02; *P* = .22). There was a trend towards increased BG PVS cluster counts in the stroke group compared to controls (exp(β) = 1.19; 95% CI, 1.04, 1.39; *P* = .019; *P_adj_* = .062). However, this association lost statistical significance upon adjustment for multiple comparisons. There were no significant between-group effects at the 36-month timepoint for BG PVS cluster counts (exp(β) = 0.93; 95% CI, 0.86, 1.02; *P* = .11).

### Lobar perivascular spaces

The stroke group exhibited significantly higher PVS volume and cluster counts in the frontal (volume: exp(β) = 1.69; 95% CI, 1.28, 2.33; *P* = .001; *P_adj_* = .009; and counts: exp(β) = 1.43; 95% CI, 1.12, 1.84; *P* = .003; *P_adj_* = .015), parietal (volume: exp(β) = 1.39; 95% CI, 1.04, 1.83; *P* = .019; *P_adj_* = .062; and counts: exp(β) = 1.69; 95% CI, 0.78, 4.95; *P* < .001; *P_adj_* < .001) and temporal lobes (volume: exp(β) = 1.6; 95% CI, 1.12, 2.31; *P* = .008; *P_adj_* = .03; and counts: exp(β) = 1.52; 95% CI, 1.12, 2.04; *P* = .005; *P_adj_* = .021) than controls. In the occipital lobe, neither PVS volumes (exp(β) = 1.61; 95% CI, 0.82, 3.12; *P* = .18) nor PVS counts (exp(β) = 1.26; 95% CI, 0.08, 21.33; *P* = .84) were significantly different between stroke patients and controls.

During the 3-year period, parietal lobe PVS volume and counts increased in controls. In contrast, the stroke group showed significant reductions in parietal lobe PVS cluster counts (exp(β) = 0.84; 95% CI, 0.68, 1.08; *P* < .001; *P_adj_* < .001) but not PVS volumes (exp(β) = 0.93; 95% CI, 0.85, 1.01; *P* = .10). No significant group-by-time interactions were observed in the temporal lobe for either PVS volume or cluster counts (all *P* > .05).

In the frontal lobe, there was a trend towards reduced PVS volumes (exp(β) = 0.9; 95% CI, 0.82, 0.99; *P* = .032; *P_adj_* = .089) and cluster counts (exp(β) = 0.91; 95% CI, 0.83, 1; *P* = .037; *P_adj_* = .092) in the stroke patients relative to controls over the 36-month period. However, these associations did not remain significant following multiple comparisons correction. In contrast, PVS metrics in the temporal (volume: exp(β) = 0.95; 95% CI, 0.86, 1.01; *P* = .10; and counts: exp(β) = 0.93; 95% CI, 0.83, 1.03; *P* = .17) and occipital lobes (volume: exp(β) = 0.9; 95% CI, 0.78, 1.06; *P* = .23; and counts: exp(β) = 1.11; 95% CI, 0.18, 4.96; *P* = .44) showed no significant difference compared to controls over the 36-month period.

### Perivascular spaces via vascular territories

The vascular territory-based analyses largely reflect the same underlying findings as the lobar analyses. This overlap arises because the MCA perfuses most cortical and subcortical regions being examined in our study. Nevertheless, we observed that PVS volume was significantly higher at baseline in the stroke group compared to controls in the ACA (exp(β) = 1.59; 95% CI, 1.17, 2.12; *P* = .003; *P_adj_* = .015) and MCA territories (exp(β) = 1.32; 95% CI, 1.1, 1.62; *P* = .004; *P_adj_* = .018), but not the PCA territory (exp(β) = 1.28; 95% CI, 0.88, 1.85; *P* = .19). In contrast, PVS cluster counts were significantly higher in the stroke group than the control group in the MCA territory (exp(β) = 1.34; 95% CI, 1.1, 1.59; *P* = .002; *P_adj_* = .014), but not the ACA territory (exp(β) = 1.36; 95% CI, 1.07, 1.75; *P* = .014; *P_adj_* = .05).

Over the 36-month period, stroke-free controls exhibited PVS enlargement whereas stroke patients exhibited a decrease in PVS cluster counts in the MCA territory (volume: exp(β) = 0.93; 95% CI, 0.87, 0.99; *P* = .023; *P_adj_* = .066; and counts: exp(β) = 0.9; 95% CI, 0.84, 0.97; *P* = .006; *P_adj_* = .024). No significant longitudinal differences in PVS metrics in the ACA vascular territory were observed between groups over the 36-month period (all *P* > .05).

### Perivascular spaces in the midbrain and hippocampus

The hippocampus is largely supplied by the PCA, whereas the midbrain is located within the posterior circulation but is not supplied by branches of the basilar artery. The logistic mixed-effects models revealed no significant group effects or longitudinal changes in midbrain or hippocampal PVS volumes or cluster counts (all *P* > .05).

## Discussion

In this longitudinal case–control study, we observed spatially distinct alterations in MRI-visible PVS in the ischaemic stroke group. The stroke group exhibited significantly enlarged PVS compared to the control group in the white matter, frontal, parietal and temporal lobes, and anterior and MCA territories. Over the study period, PVS counts and volumes increased in the control group, consistent with age-related enlargement. Contrary to our hypothesis, stroke group PVS volume and counts reduced over the 36-month period, with the most pronounced reductions observed in the parietal lobe and MCA territory, compared to baseline. Together, the findings indicate that ischaemic stroke induces region-specific changes in PVS, with stroke patients showing a reduction in PVS within the first 3 years post-stroke.

Previous studies investigating PVS in stroke populations have yielded inconsistent results, often using semi-quantitative 2D rating scales.^34^ Enlarged BG PVS have been identified in 2 studies as a risk factor for incident ischaemic stroke,^8^^,^^35^ but an association was found between BG PVS and lacunar ischaemic stroke only in another study.^36^ A meta-analysis reported significant associations between PVS burden and ageing, hypertension and lacunes, but found no direct association with stroke.^32^ In contrast, Ballerini et al. (2020) applied computational methods to quantify PVS in community-dwelling individuals and observed significantly increased PVS width and size in participants with recurrent stroke.^37^

Our finding of a longitudinal reduction in PVS counts and volume in stroke survivors is novel. This observation was made possible by our longitudinal study design, which followed patients over a 3-year period, and has implications for the interpretation of MRI-perivascular space measurements. Currently, the MRI-PVS enlargement is regarded as a biomarker of CSVD.^7^ Consistent with this idea, our stroke group exhibited a significantly larger PVS burden than controls; however, the longitudinal PVS trajectories progressed in opposite directions between the 2 groups: over the 3-year study period, PVS continued to enlarge in controls, whereas stroke survivors demonstrated significant shrinkage in multiple brain regions.

Enlarged PVS in stroke patients may reflect pathological processes preceding the ischaemic event or may instead result from the stroke itself. For example, hyperacute enlargement of PVS can be driven by mechanisms such as impaired glymphatic clearance, blood–brain-barrier dysfunction and inflammatory cell infiltration.^12^^,^^38^ Our study design does not allow us to determine whether PVS changes precede stroke, either as a causal factor or epiphenomenon, or whether they arise acutely as a consequence of the stroke event. Another possible explanation is that local atrophy and Wallerian degeneration contributed to the gradual reduction in PVS observed over time.

Nevertheless, our finding of PVS shrinkage 3 years after ischaemic stroke supports the notion that PVS volume and count trajectories are not linear,^10^ and that PVS morphology alone may not serve as a readily interpretable biomarker of brain health. It is possible that accelerated brain atrophy, which we previously reported in this cohort^4^^,^^22^ would have contributed to structural collapse of white matter, thereby reducing the volume and visibility of PVS.

Perivascular spaces are typically studied in the white matter or BG. One study in healthy adults reported a greater prevalence of PVS within the ACA and MCA territories than the PCA territory.^39^ Theoretically, stroke-related alterations in perfusion, vascular integrity and glymphatic function may differentially affect PVS across vascular territories, hence our exploration in this study. We observed longitudinal changes in PVS burden within the MCA territory but not the ACA or PCA territories. We note that the MCA territory captures more of the white matter and subcortical regions, which may in part explain this association. Perivascular spaces are seen in white matter regions which are largely perfused by deep penetrating vessels, mostly from the MCA. This may be a function of anatomy rather than true differences in haemodynamic or perfusion characteristics between vascular territories. In addition, anterior circulation stroke predominantly involves the MCA, not ACA and is the commonest territory affected. Furthermore, we found no significant associations between stroke diagnosis and PVS burden in the midbrain or hippocampus. The hippocampus is a largely PCA-supplied region, and the midbrain is supplied by posterior circulation (basilar) branches below the circle of Willis arteries.^40^ Enlarged PVS in the midbrain has been linked to Parkinson’s disease and cerebrospinal fluid levels of tau protein.^41^ In a previous study, we observed significant ipsilesional atrophy of hippocampal subfields in ischaemic stroke patients when assessed at the 12-month follow-up.^42^ Given the small sample size and lower prevalence of detectable PVS in midbrain and hippocampi, larger cohorts may be necessary to detect subtle PVS changes in these regions.

Our findings demonstrate that PVS trajectories are dynamic following stroke and vary across brain regions. Although PVS were enlarged in stroke patients at baseline, they gradually decreased over the 3 years after stroke, most notably in the parietal lobe. These findings suggest that MRI-visible PVS may reflect post-stroke tissue remodelling and highlight the necessity of longitudinal assessments, rather than interpretation from a single timepoint. However, further studies are required to determine whether longitudinal PVS trajectories are predictive of patient outcomes.

### Strengths and limitations

Strengths of this study include the use of a longitudinal design and a novel validated deep learning approach for automated PVS quantification. However, several limitations should be acknowledged. First, our cohort was restricted to patients with ischaemic stroke, limiting generalisability to other stroke subtypes such as haemorrhagic stroke. Second, stroke heterogeneity, including variation in lesion size, location, arterial territory, aetiology and side could have introduced variability. Consequently, region-based analyses may further be underpowered to detect regional effects. We did not investigate whether the stroke side or site influenced the spatiotemporal evolution of MRI-visible PVS. Future studies should investigate the influence of stroke lesion location and volume on region-specific longitudinal changes in PVS, while accounting for the potential confounding effects of local atrophy and Wallerian degeneration. Furthermore, we did not examine associations between PVS burden and other markers of CSVD, such as WMH, lacunar infarctions or cerebral microbleeds. The presence of WMH can obscure the visibility of PVS in MRI,^43^ and previous studies have reported that microbleeds often co-occur with enlarged PVS.^44^^,^^45^ Characterising these CSVD markers would have enabled a more comprehensive comparison of CSVD burden between the control and stroke cohorts. Future longitudinal studies integrating PVS with established markers of CSVD are needed to clarify the mechanisms underlying post-stroke PVS changes.

Finally, PVS were segmented from T1w MRI, which is inherently less sensitive for PVS detection compared to T2w sequences using current segmentation methods. Although the PVS segmentation model demonstrated robust performance on MRI data from the study cohort,^20^ external validation in independent datasets is still required, alongside replication of these findings in other ischaemic stroke cohorts to establish their generalisability. Future studies investigating PVS using T1w MRI should consider validating PVS measurements with T2w MRI, where available, given its greater sensitivity for PVS detection.

## Conclusion

We found that stroke survivors exhibited greater PVS counts and volumes compared to stroke-free controls at the 3-month timepoint following stroke. However, while the control group exhibited an expected age-related increase in PVS measurements over the 3-year follow-up period, our stroke group exhibited reductions in PVS counts and volumes in several brain regions. In summary, we provide novel evidence that perivascular space alterations are dynamic over the 3 years after ischaemic stroke. While these observations highlight the potential of MRI-PVS as an imaging biomarker of post-stroke outcomes, further research is necessary to clarify their biological underpinnings and determine the clinical contexts in which PVS measurements could be most effectively applied.

## Supplementary Material

Supplementary_material_aakag108

## Data Availability

The dataset analysed in the current study is available from the corresponding author upon reasonable request.
