# Supplementary material for: Tracking the changes in longitudinal MRI-detected perivascular spaces following ischaemic stroke
Source: Eur Stroke J. 2026 Sep 7;11(9):aakag108. doi: 10.1093/esj/aakag108 (PMC13547861; doi:10.1093/esj/aakag108)
Supplement: Supplementary file 1 [file supplementary_material_aakag108.zip › Supplementary_Table_1_aakag108.docx]

**Supplementary Table 1.** Descriptive statistics of manually counted perivascular spaces on baseline MRI scans. PVS counts in the centrum semiovale (CS) and basal ganglia (BG) were obtained from two representative axial slices, with descriptive statistics based on the slice containing the higher PVS count. PVS counts in the midbrain (MB) and hippocampus (HP) were obtained from the entire anatomical region. Values are presented as median [Q1, Q3]. Differences between groups were assessed with Wilcoxon rank-sum tests.

| **Region** | **Control (*n*=39)** | **Stroke (*n*=124)** | **Total (*N*=163)** | ***p*-value** |
| --- | --- | --- | --- | --- |
| **CS** | 13 [10, 19] | 14 [9, 21] | 14 [9, 20.5] | 0.78 |
| **BG** | 10 [7.5, 13] | 10 [7, 14] | 10 [7, 13] | 0.95 |
| **MB** | 4 [3, 5.5] | 3 [2, 4.3] | 3 [2, 5] | 0.04 |
| **HP** | 2 [1, 4] | 3 [1, 4] | 3 [1, 4] | 0.18 |
