# Supplementary material for: Tracking the changes in longitudinal MRI-detected perivascular spaces following ischaemic stroke
Source: Eur Stroke J. 2026 Sep 7;11(9):aakag108. doi: 10.1093/esj/aakag108 (PMC13547861; doi:10.1093/esj/aakag108)
Supplement: Supplementary file 1 [file supplementary_material_aakag108.zip › Supplementary_Table_2_v2_aakag108.docx]

**Supplementary Table 2.** Correlation and concordance between manual and model-predicted perivascular space counts in the centrum semiovale (CS), basal ganglia (BG), midbrain (MB), and hippocampus (HP). ρ=Spearman’s rho; CCC=Lin’s concordance correlation coefficient; TP=timepoint.

| **Region** | **Group** | **TP** | **Spearman's ρ**  **(*p* < 0.01)** | **CCC** | **95%CI** |
| --- | --- | --- | --- | --- | --- |
| CS | All | Baseline | 0.88 | 0.89 | 0.87–0.91 |
| CS | All | 12M | 0.86 | 0.79 | 0.75–0.82 |
| CS | All | 36M | 0.84 | 0.77 | 0.74–0.81 |
| CS | Control | Baseline | 0.88 | 0.93 | 0.89–0.95 |
| CS | Control | 12M | 0.84 | 0.85 | 0.8–0.9 |
| CS | Control | 36M | 0.85 | 0.84 | 0.78–0.88 |
| CS | Stroke | Baseline | 0.89 | 0.88 | 0.85–0.91 |
| CS | Stroke | 12M | 0.86 | 0.76 | 0.71–0.79 |
| CS | Stroke | 36M | 0.85 | 0.74 | 0.69–0.78 |
| BG | All | Baseline | 0.73 | 0.72 | 0.67–0.77 |
| BG | All | 12M | 0.78 | 0.67 | 0.62–0.72 |
| BG | All | 36M | 0.74 | 0.67 | 0.61–0.73 |
| BG | Control | Baseline | 0.8 | 0.79 | 0.69–0.85 |
| BG | Control | 12M | 0.81 | 0.66 | 0.55–0.74 |
| BG | Control | 36M | 0.75 | 0.6 | 0.5–0.69 |
| BG | Stroke | Baseline | 0.71 | 0.7 | 0.64–0.76 |
| BG | Stroke | 12M | 0.77 | 0.68 | 0.61–0.74 |
| BG | Stroke | 36M | 0.74 | 0.69 | 0.62–0.76 |
| MB | All | Baseline | 0.53 | 0.52 | 0.4–0.62 |
| MB | All | 12M | 0.65 | 0.48 | 0.39–0.57 |
| MB | All | 36M | 0.61 | 0.48 | 0.37–0.57 |
| MB | Control | Baseline | 0.59 | 0.57 | 0.33–0.74 |
| MB | Control | 12M | 0.63 | 0.54 | 0.32–0.71 |
| MB | Control | 36M | 0.45 | 0.25 | 0.04–0.44 |
| MB | Stroke | Baseline | 0.47 | 0.49 | 0.35–0.61 |
| MB | Stroke | 12M | 0.62 | 0.46 | 0.36–0.56 |
| MB | Stroke | 36M | 0.71 | 0.57 | 0.45–0.67 |
| HP | All | Baseline | 0.69 | 0.67 | 0.59–0.74 |
| HP | All | 12M | 0.72 | 0.67 | 0.58–0.75 |
| HP | All | 36M | 0.73 | 0.66 | 0.57–0.74 |
| HP | Control | Baseline | 0.54 | 0.57 | 0.35–0.72 |
| HP | Control | 12M | 0.63 | 0.72 | 0.54–0.84 |
| HP | Control | 36M | 0.72 | 0.78 | 0.61–0.88 |
| HP | Stroke | Baseline | 0.72 | 0.69 | 0.59–0.76 |
| HP | Stroke | 12M | 0.73 | 0.65 | 0.54–0.74 |
| HP | Stroke | 36M | 0.72 | 0.63 | 0.51–0.72 |
